# Physician Characteristics Associated with Prioritizing Treatment Burden in Outpatient Care for Older Adults with Multimorbidity

**DOI:** 10.31662/jmaj.2025-0452

**Published:** 2025-11-21

**Authors:** Takuma Kimura, Ken Shinmura, Shinji Matsumura, Masayoshi Hashimoto

**Affiliations:** 1 Department of R&D Innovation for Home Care Medicine, Department of General Medicine, Institute of Science Tokyo, Tokyo, Japan; 2 Department of General Internal Medicine, Hyogo Medical University, School of Medicine, Hyogo, Japan; 3 Matsumura Clinic/Division of Clinical Epidemiology, National Hospital Organization Tokyo Medical Center, Tokyo, Japan

**Keywords:** multimorbidity, older adults, treatment burden, geriatricians, outpatients, primary care

## Abstract

**Introduction:**

Older adults with multimorbidity often experience substantial treatment burden, which can compromise their quality of life and adherence to medical treatment. Accordingly, physicians need to prioritize treatment burden. However, physician characteristics associated with prioritization of treatment burden among older adults with multimorbidity in outpatient care remain unclear. This study aimed to: (1) develop a brief, clinician-oriented scale to assess prioritization of treatment burden in outpatient settings and evaluate its reliability and exploratory validity; and (2) examine associations between prioritizing treatment burden and physician attributes in Japan.

**Methods:**

We conducted an anonymous postal survey in June and July 2022 targeting 3,300 physicians affiliated with the Japan Geriatrics Society or the Japan Primary Care Association. Physicians’ prioritization of treatment burden was assessed using a newly developed six-item scale. After evaluating the scale’s reliability and validity, we dichotomized participants by the median score and used modified Poisson regression to analyze associations between prioritizing treatment burden and physician characteristics (sex, years of experience, and practice setting).

**Results:**

Responses from 688 physicians who provided outpatient care were analyzed. The scale demonstrated good construct validity and internal consistency (Cronbach’s alpha = 0.771). Female physicians were significantly more likely to prioritize treatment burden than male physicians (model 1: prevalence ratio [PR] 1.204, 95% confidence interval [CI]: 1.084-1.336; model 2: PR 1.202, 95% CI: 1.082-1.335). No significant associations were found between prioritizing treatment burden and years of clinical experience or practice environment (e.g., facility type, patient age distribution).

**Conclusions:**

The newly developed clinician-oriented scale demonstrated exploratory validity for assessing physicians’ prioritization of treatment burden. Furthermore, prioritization of treatment burden was more strongly associated with personal attributes―particularly sex―than with clinical experience or practice setting. These findings underscore the need for educational interventions to enhance physicians’ awareness of treatment burden.

## Introduction

Japan is the most rapidly aging country in the world, and the proportion of older adults with multimorbidity is expected to increase. In such patients, the cumulative demands of medication management, clinic visits, self-care, and lifestyle modifications constitute a substantial treatment burden, which can lead to poor medical treatment adherence, treatment discontinuation, and ultimately impaired disease control and adverse health outcomes ^(1), (2), (3)^. Older adults are particularly vulnerable to treatment burden because of cognitive decline, impaired activities of daily living, and limited social support, which may compromise both their quality of life (QOL) and the quality of care they receive ^(4), (5), (6), (7)^.

Japan’s healthcare system is characterized by a strong reliance on outpatient services, and has one of the highest rates of outpatient visits among Organisation for Economic Co-operation and Development (OECD) countries ^(8)^. Consequently, clinic visits often become a significant component of treatment burden ^(3)^. The highly specialized nature of Japanese healthcare also means many older adults with multimorbidity must attend multiple departments, which further compounds treatment burden. Therefore, outpatient care for older adults with multimorbidity in Japan is structurally prone to generating treatment burden.

Previous studies showed that physicians tended to underestimate patients’ treatment burden, leading to a mismatch between physicians’ perceptions and patients’ subjective experiences of daily challenges, medication management, and clinic visits ^(9), (10)^. This gap may contribute to reduced treatment adherence and fragmented care. Treatment burden should be evaluated from the patient’s perspective, but cognitive decline may make self-assessment difficult among older adults ^(5), (7)^. Therefore, in addition to caregiver input, physicians require a framework that enables them to recognize and actively respond to treatment burden in clinical practice, particularly in time-constrained outpatient settings. However, no simple scale currently exists for physicians to directly assess treatment burden. We hypothesized that physicians may find it more intuitive to understand treatment burden when framed in terms of QOL decrements caused by treatment itself.

In Western countries, general practitioners and geriatricians are increasingly recognized as playing central roles in alleviating treatment burden in outpatient care for older adults with multimorbidity ^(11), (12), (13)^. A similar role is expected in Japan. However, little is known about the extent to which physicians in Japan prioritize treatment burden, or how this prioritization relates to physician attributes such as sex, years of clinical experience, and practice setting. Identifying these characteristics may be crucial for designing educational programs and policy interventions to improve the quality of care for older adults with multimorbidity.

This study had two aims. First, we developed a brief, clinician-oriented scale to enable physicians to conceptualize treatment burden in relation to QOL and evaluated its reliability and exploratory validity. Second, we examined how physicians providing outpatient care in Japan perceived treatment burden in older adults with multimorbidity and explored associations between prioritization of treatment burden and physician attributes, including sex, clinical experience, and practice environment.

## Materials and Methods

### Study population

We conducted an anonymous, mail-based questionnaire survey in June and July 2022. To specifically target physicians who are extensively involved in the outpatient care of older adults with multimorbidity, we included all 1,650 certified geriatricians registered with the Japan Geriatrics Society as of April 2022. In addition, to capture physicians providing outpatient care for older adults with multimorbidity in community settings who were not organ- or disease-specific specialists, we randomly selected 1,650 physicians from among the 6,526 eligible members of the Japan Primary Care Association (JPCA). Eligible JPCA members comprised 1,091 board-certified family medicine specialists and 5,435 board-certified primary care physicians. In total, 3,300 physicians were included in our study sample.

### Questionnaire

The questionnaire comprised 15 items, including the primary outcome measure (i.e., prioritization of treatment burden in the outpatient care of older adults with multimorbidity), participant characteristics, and items used for other research purposes ^(14), (15)^.

#### Analytical model

Our literature review revealed no prior studies examining the relationship between physician attributes and their awareness of treatment burden. Based on previous literature, we hypothesized that attributes associated with higher prioritization of treatment burden would be: (1) more years of experience, (2) female sex (women were previously shown to be more patient-centered), (3) practicing in smaller municipalities, (4) frequently caring for patients aged ≥90 years, and (5) working in clinics, home visit settings, or long-term care facilities ^(4), (5), (6), (7), (9), (16), (17), (18)^. We restricted our analysis to physicians engaged in outpatient practice because outpatient care is a key setting for managing older adults with multimorbidity and delivering care in resource- and time-constrained environments.

#### Questions on prioritization of treatment burden

We developed a six-item scale to assess the extent to which physicians prioritized treatment burden based on prior patient-reported burden scales ^(19), (20), (21)^ and internal discussions. We reframed the concept of treatment burden using QOL as a lens to enhance physician understanding and included both disease management and logistical aspects of care. The final items were: “Dietary therapy reduces QOL,” “Exercise therapy reduces QOL,” “Pharmacotherapy reduces QOL,” “Waiting time is burdensome,” “Visiting outpatient clinics is burdensome,” and “Overall treatment burden exists.” Each item was scored on a 4-point Likert scale from 1 (not prioritized at all) to 4 (highly prioritized).

The reliability and validity of the scale was evaluated. We assessed construct validity by calculating Pearson’s correlation coefficients for convergent validity between our scale and two theoretically relevant items: “Assessing patient burden” and “Revising treatment plans.” To evaluate the discriminant validity, we used two items expected to be weakly correlated with treatment burden prioritization: “difficulty addressing social issues” and “difficulty treating patients with severe comorbidities.”

We conducted exploratory factor analysis (EFA) to examine latent factor structures. Principal component analysis was used for extraction and varimax rotation and Kaiser normalization was applied. Factor retention was based on eigenvalues >1.0 (Kaiser criterion) and visual inspection of the scree plot. Cronbach’s alpha was calculated to assess internal consistency.

#### Physician characteristics

Participants were asked to report their sex, age, and clinical experience (years and months). Practice setting was recorded as clinic without beds, clinic with beds, hospital with <200 beds, hospital with ≥200 beds, tertiary care hospital, or long-term care/older adult care facility. Participants also reported their practice type (outpatient, home visits, long-term care, inpatient) and municipality population size (<50,000, 50,000-100,000, 100,000-300,000, 300,000-500,000, >500,000). Frequency of care by patient age group (65-74 years, 75-89 years, ≥90 years) was assessed using four categories: “never,” “rarely,” “sometimes,” and “frequently.”

### Data analysis

#### Treatment burden prioritization

Items were scored from 1 to 4 points, and these scores were summed to calculate a total score from 6 to 24. Higher scores reflected greater prioritization of treatment burden. Participants were classified into two groups based on the median score: those scoring above the median were assigned to the “high prioritization” group; those scoring below the median to the “low prioritization” group.

#### Background characteristics

Municipality size was dichotomized as <100,000 (non-urban) and ≥100,000 (urban) based on previous studies. Frequency of treating patients in each age group was categorized as “low” (never or rarely) or “high” (sometimes or frequently). We compared participants’ background characteristics across prioritization groups using chi-square tests, Fisher’s exact tests, or Mann-Whitney U-tests, as appropriate.

#### Factors associated with prioritization

We used modified Poisson regression to calculate prevalence ratios (PRs) and 95% confidence intervals (CIs), with high prioritization ^(1)^ versus low prioritization (0) as the outcome ^(22)^. Covariates included years of experience (continuous), sex (male/female), municipality population size (<100,000/≥100,000), and frequency of caring for patients aged ≥90 years (high/low). Model 1 included these covariates plus facility type (clinic/hospital). Model 2 further included inpatient care, home visits, and facility-based care. We assessed multicollinearity using variance inflation factors (VIFs) with a threshold of <10. All analyses were two-tailed, with the significance level set at p < 0.05. Statistical analyses were performed using IBM SPSS Statistics for Windows, Version 29.0.1 (IBM Corp., Armonk, NY, USA).

### Ethical approval

This study was approved by the Ethics Committee of the Maruki Memorial Medical and Social Welfare Center (Approval Number 37). Consent was obtained through the voluntary return of completed questionnaires, which included a cover sheet describing the study’s aims, data protection procedures, and researcher contact information.

## Results

**Figure 1.**
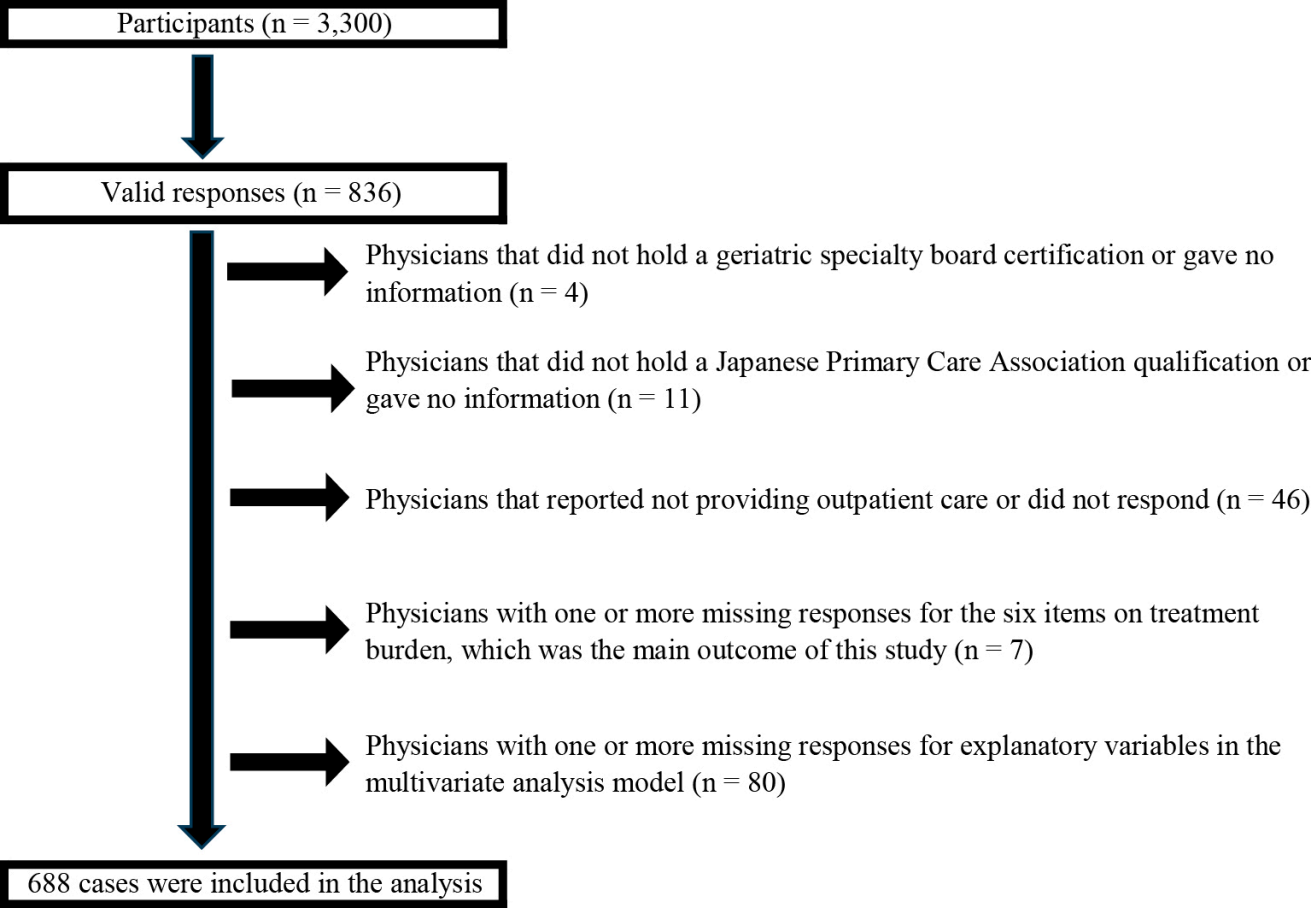
Population flow diagram of study participants.

In total, 836 responses were received (response rate 25.3%). Of these, 15 physicians were excluded: four from the geriatrics group who were not certified geriatricians and 11 from the primary care group who had neither family medicine nor primary care certification. An additional 46 physicians were excluded because they did not provide outpatient care, and seven were excluded because of incomplete responses on any of the six primary outcome items. Finally, 80 physicians were excluded because of missing data on explanatory variables. The final analytic sample comprised 688 physicians (Figure 1).

Participants’ mean age was 53.41 ± 11.89 years, and their mean clinical experience was 27.68 ± 11.63 years. The majority (n = 564, 82.0%) of participants were male and 124 (18.0%) were female.

1) Reliability and Validity of the Treatment Burden Prioritization Scale

Evaluation of convergent validity showed the Treatment Burden Prioritization Scale score was moderately correlated with “assessing patient burden” (r = 0.455) and “revising treatment plans” (r = 0.396), which was consistent with theoretical assumptions. Evaluation of discriminant validity showed that correlations with “difficulty addressing social issues” (r = 0.061) and “difficulty treating patients with severe comorbidities” (r = 0.029) were low, as expected. These results supported the convergent and discriminant validity of our scale.

EFA of the Treatment Burden Prioritization Scale indicated a two-factor structure based on eigenvalues and scree plot examination (Table 1). Items related to diet, exercise, and medication loaded on Factor 1 (“QOL impact”), and waiting time and transportation burden loaded on Factor 2 (“Outpatient visit burden”). The item “There is treatment burden” showed high cross-loading on both factors, suggesting a general factor nature. The Kaiser-Meyer-Olkin measure was 0.735 and Bartlett’s test of sphericity was significant (p < 0.001), indicating the scale had adequate factorability. The Cronbach’s alpha was 0.790 for Factor 1 (three items), 0.728 for Factor 2 (two items), 0.757 for five items combined, and 0.771 for all six items.

**Table 1.** Exploratory Factor Analysis of the Treatment Burden Prioritization Scale.

	Component 1	Component 2
Dietary therapy reduces QOL	0.876	0.104
Exercise therapy reduces QOL	0.832	0.073
Pharmacotherapy reduces QOL	0.734	0.280
Waiting time is burdensome	0.126	0.860
Visiting outpatient clinics is burdensome	0.160	0.856
Overall treatment burden exists	0.460	0.394

QOL: quality of life.

These results indicated the scale was a valid tool for assessing physician prioritization of treatment burden in the care of older adults with multimorbidity, with a clear conceptual structure and theoretical consistency.

2) Treatment Burden in the Management of Older Adults with Multimorbidity (Table 2)

**Table 2.** Survey Items on Treatment Burden for the Management of Older Adults with Multimorbidity (N = 688).

	Not prioritized at all		Minimally prioritized		Moderately prioritized		Highly prioritized	
	n	%	n	%	n	%	n	%
Dietary therapy reduces QOL	7	1.0%	121	17.6%	445	64.7%	115	16.7%
Exercise therapy reduces QOL	14	2.0%	175	25.4%	427	62.1%	72	10.5%
Pharmacotherapy reduces QOL	1	0.1%	66	9.6%	435	63.2%	186	27.0%
Waiting time is burdensome	6	0.9%	146	21.2%	452	65.7%	84	12.2%
Visiting outpatient clinics is burdensome	1	0.1%	45	6.5%	465	67.6%	177	25.7%
Overall treatment burden exists	2	0.3%	22	3.2%	341	49.6%	323	46.9%

QOL: quality of life.

Overall “moderately prioritized” was the most common response for all six items. On an item level, the most common response for “Pharmacotherapy reduces QOL,” “Visiting outpatient clinics is burdensome,” and “Overall treatment burden exists” was “highly prioritized” followed by “moderately prioritized.” For “Dietary therapy reduces QOL,” “Exercise therapy reduces QOL,” and “Waiting time is burdensome,” the most common response was “minimally prioritized” followed by “moderately prioritized.” The overall mean score for the prioritization scale was 18.47 ± 2.43 (out of 24). The median score was 19, and 193 physicians (28.1%) were classified as the low prioritization group and 495 (71.9%) as the high prioritization group.

3) Basic Characteristics and Treatment Burden Prioritization (Table 3)

**Table 3.** Basic Characteristics and Treatment Burden Prioritization (N = 688).

	Total (N=688)		High prioritization group (n = 495, 71.9%)		Low prioritization group (n =193, 28.1%)		p-Value
	n or mean	% or SD	n or mean	% or SD	n or mean	% or SD	
Sex							0.002
Male	564	82.0%	392	79.2%	172	89.1%	
Female	124	18.0%	103	20.8%	21	10.9%	
Age (years)	53.4	11.9	53.3	11.9	53.4	11.9	0.908
Experience as a physician (years)	27.7	11.6	27.7	11.5	27.7	11.7	0.929
Type of facility							0.755
Clinic	220	32.0%	160	32.3%	60	31.1%	
Hospital	468	68.0%	335	67.7%	133	68.9%	
Type of facility							0.935
Non-bedded clinic	199	28.9%	53	27.5%	146	29.5%	
Bedded clinic	21	3.1%	7	3.6%	14	7.0%	
Hospital with <200 beds	170	24.7%	46	23.8%	124	25.1%	
Hospital with ≥200 beds	212	30.8%	61	31.6%	151	30.5%	
University hospital	86	12.5%	26	13.5%	60	12.1%	
Clinical setting							
Ward							0.386
Provided	374	54.4%	264	53.3%	110	57.0%	
Not provided	314	45.6%	231	46.7%	83	43.0%	
Home medical care							0.269
Provided	265	38.5%	197	39.8%	68	35.2%	
Not provided	423	51.5%	298	60.2%	125	64.8%	
Long-term care facility							0.144
Provided	184	26.7%	140	28.3%	44	22.8%	
Not provided	504	73.3%	355	71.7%	149	77.2%	
Municipality population size							0.628
Under 100,000	187	27.2%	132	26.7%	55	28.5%	
Over 100,000	501	72.8%	363	73.3%	138	71.5%	
Frequency of treating patients aged 65-75 years							0.484
Low	2	0.3%	1	0.2%	1	0.5%	
High	684	99.7%	492	99.8%	192	99.5%	
Frequency of treating patients aged 75-89 years							0.192
Low	3	0.4%	1	0.2%	2	1.0%	
High	684	99.6%	493	99.8%	191	99.0%	
Frequency of treating patients aged ≥90 years							0.324
Low	37	5.4%	24	4.8%	13	6.7%	
High	651	94.6%	471	95.2%	180	93.3%	

SD: standard deviation. Missing values: Sex (n = 0), Age (n = 8), Experience as a physician (n = 0), Type of facility (n = 0), Clinical setting (n = 0), Population size of the municipality (n = 0). Frequency of treating patients aged 65-75 years (n = 2), Frequency of treating patients aged 75-89 years (n = 1), Frequency of treating patients aged ≥90 years (n = 0).

Female physicians were significantly more likely to be in the high prioritization group than male physicians (p < 0.001). No significant differences were observed between treatment burden prioritization and age or years of clinical experience. There were no statistically significant differences between prioritization groups for types of clinical service (e.g., home visits, long-term care facility care, inpatient care), municipality population size, or frequency of treating patients by age group (65-74 years, 75-89 years, ≥90 years).

4) Factors Associated with the Prioritization of Treatment Burden in Outpatient Care for Older Patients (Table 4)

**Table 4.** Factors Associated with the Prioritization of Treatment Burden in Outpatient Care for Older Patients Among Geriatricians and General Practitioners (N = 688).

	Model 1				Model 2			
	Prevalence ratio	95% confidence interval		p-Value	Prevalence ratio	95% confidence interval		p-Value
		Lower	Higher			Lower	Higher	
Experience as a physician (years)	1.002	0.997	1.006	0.461	1.002	0.997	1.006	0.461
Sex								
Female	1.204	1.084	1.336	<0.001	1.202	1.082	1.335	<0.001
Male	REF				REF	-	-	
Municipality population size								
Under 100,000	0.987	0.887	1.098	0.808	0.978	0.878	1.089	0.679
Over 100,000	REF	-	-		REF	-	-	
Frequency of treating patients aged ≥90 years								
High	1.111	0.866	1.425	0.408	1.094	0.852	1.404	0.481
Low	REF	-	-		REF	-	-	
Type of facility								
Clinic	1.011	0.916	1.116	0.828	-	-	-	
Hospital	REF	-	-		-	-	-	
Clinical setting								
Ward								
Provided	-	-	-		0.969	0.880	1.066	0.518
Not provided	-	-	-		REF	-	-	
Home medical care								
Provided	-	-	-		1.019	0.906	1.146	0.754
Not provided	-	-	-		REF	-	-	
Long-term care facility								
Provided	-	-	-		1.067	0.947	1.201	0.286
Not provided	-	-	-		REF	-	-	

REF: reference.

In both model 1 (PR 1.204, 95% CI: 1.084-1.336) and model 2 (PR 1.202, 95% CI: 1.082-1.335), being female was significantly associated with higher prioritization of treatment burden compared with being male. Kendall correlation coefficients among independent variables showed no multicollinearity (τ < 0.70, maximum was 0.207 in model 1 and 0.393 in model 2). The VIFs were ≤1.078 in model 1 and ≤1.499 in model 2.

## Discussion

This study focused on physicians’ prioritization of treatment burden in the provision of outpatient care of older adults with multimorbidity. First, we demonstrated that the newly developed six-item scale for assessing treatment burden prioritization showed reliability and exploratory validity. We then clarified the associations between prioritization of treatment burden and physician characteristics and practice environments in the context of outpatient care. To our knowledge, few previous studies evaluated physicians’ attitudes toward treatment burden from this perspective, meaning our study is a pioneering attempt in this field.

The six-item scale developed in this study was structured around two conceptual domains: “impact of treatment on QOL” and “burden of outpatient visits.” This framework allowed for a brief, practical assessment across multiple domains, including treatment modalities, lifestyle modifications, and access to care. Previous research showed that discrepancies often exist between patients’ self-perceived treatment burden and physicians’ understanding thereof, with physicians tending to focus predominantly on medical factors ^(9), (10), (23)^. Our scale may help bridge this perceptual gap in geriatric and primary care settings when used alongside existing patient-reported measures, thereby serving as a useful tool for shared decision-making, including treatment adjustment and deprescribing ^(24)^. From a practical perspective, our scale can be readily integrated into outpatient workflows because it can be quickly completed by physicians before or after patient encounters. Scores above a certain threshold may serve as a cue to reconsider treatment strategies, but the scale may be particularly useful when scores fall below a set threshold. As all patients with multimorbidity experience some degree of treatment burden, low scores could function as a reminder for physicians to heighten their awareness of this issue ^(1), (3), (4)^. For example, based on the scale items, such reminders may prompt physicians to reconsider specific clinical actions, including deprescribing, modifying lifestyle recommendations, or adjusting follow-up intervals. Furthermore, when discrepancies arise between physicians’ scores and patient-reported measures, comparison of these results may stimulate dialogue, uncover hidden patient concerns, and ultimately contribute to more individualized and patient-centered care.

A key finding of this study was that sex was significantly associated with physicians’ prioritization of treatment burden. Female physicians were more likely to prioritize treatment burden than male physicians. This suggested that female physicians may be more sensitive to the impact of treatment on patients’ daily lives and the logistical burdens of outpatient visits. This result was consistent with previous studies that reported female physicians were more likely to adopt patient-centered approaches than male physicians ^(16), (17), (18)^. Female physicians are also known to engage in empathetic communication, employ holistic perspectives, spend more time per consultation, and devote attention to psychosocial contexts ^(17), (18)^. These factors may contribute to their heightened awareness of treatment burden. The sex difference observed in this study may reflect social and cultural gender roles in addition to biological sex differences ^(17), (25)^.

In contrast, clinical experience, engagement in home or facility-based care, and frequency of caring for different age groups of older adults were not significantly associated with prioritization of treatment burden. Several interpretations for these findings are possible. First, extensive clinical experience does not necessarily translate into a deeper recognition of treatment burden. Without structured education or opportunities for continuing learning, it may be difficult for physicians to acquire awareness of treatment burden through experience alone. Second, even when physicians regularly care for the oldest old or practice in long-term care settings, a predominant focus on medical management may limit recognition of patients’ subjective burdens and psychosocial needs. Previous studies suggested that clinics may provide higher-quality care for older adults with multimorbidity compared with hospitals ^(26)^. However, our study found no significant association between practice setting and prioritization of treatment burden. This suggested physicians’ individual attitudes and values may play a greater role than practice environment in shaping their recognition of treatment burden. Educational interventions therefore hold potential to effect meaningful change. Although recent educational initiatives in Japan have begun to address outpatient care for patients with multimorbidity, training specifically focused on treatment burden remains lacking ^(27)^. Developing educational programs that explicitly integrate treatment burden into outpatient care for older adults is essential.

This study had several limitations. First, although the scale we developed demonstrated construct validity and internal consistency, its content validity remains exploratory. Further research should assess our scale’s agreement with patients’ self-reported treatment burden, compare it with existing patient-reported measures, and examine concordance or discordance with caregiver-reported burden, particularly among older patients with cognitive impairment. Reproducibility across multiple sites should also be evaluated ^(24)^. Second, this study targeted geriatricians and certified physicians from the JPCA, who represent physicians providing outpatient care for older adults with multimorbidity but not organ- or disease-specific specialists. Although this sampling strategy provided a degree of representativeness, participation was voluntary, which raises the possibility of selection bias if physicians with stronger interest in treatment burden were most likely to respond. Third, the survey relied on self-reported data, which may not fully reflect actual clinical behaviors. Fourth, physician sex was analyzed as a binary variable (male/female). The observed sex differences may partly reflect broader gender roles. Further studies should examine how sex and gender perspectives influence recognition and management of treatment burden in greater detail ^(17), (28)^.

The complexity of multiple coexisting conditions renders additive treatment approaches based solely on single-disease guidelines insufficient for older adults with multimorbidity ^(12), (13)^. Because additive approaches may exacerbate treatment burden, clinicians must not only decide “what to treat,” but also determine “which treatments burden patients most,” prioritize accordingly, and consider discontinuing therapies when appropriate. Raising physicians’ awareness of treatment burden is clinically and policy-relevant. Alleviating treatment burden may improve patients’ QOL and treatment adherence, and reduce avoidable hospitalizations and healthcare costs. Therefore, widespread use of the six-item Treatment Burden Prioritization Scale in daily practice could foster more patient-centered multimorbidity care in Japan.

Our findings suggest that physicians’ sex has a greater influence on attitudes toward treatment burden than clinical experience or practice environment. This implies that awareness of treatment burden may not be naturally acquired through experience alone. Rather, structured educational interventions are needed to enhance physicians’ recognition of treatment burden. Developing training programs focused on treatment burden―including its assessment, shared decision-making, and deprescribing―may improve the quality of care for older adults with multimorbidity. Incorporating such modules into residency training, continuing professional development, and board certification for geriatric and primary care specialists could help shift physician attitudes and contribute to overall improvement in care quality.

## Article Information

### Acknowledgments

We express our deep appreciation to Dr. Masahiro Akishita, former President of the JGS, geriatric specialists; Dr. Tetsushu Kusaba, President of the JPCA, family medicine specialists; and primary care certified physicians for their cooperation with this survey.

### Author Contributions

Takuma Kimura and Ken Shinmura conceived the research questions. Takuma Kimura and Ken Shinmura contributed to the design of the research protocol and the development of the questionnaire. Takuma Kimura and Shinji Matsumura conducted the statistical analysis. Takuma Kimura drafted the initial manuscript in collaboration with Ken Shinmura, Shinji Matsumura, and Masayoshi Hashimoto. All authors revised and approved the final manuscript.

### Conflicts of Interest

First author reports receiving research grants from CUC (Change Until Change) Inc. and Kyouseikai Medical Corporation, Japan. The other authors declare that there are no conflicts of interest.
